# A case report of malignant hyperthermia in a patient with myotonic dystrophy type I: A CARE-compliant article

**DOI:** 10.1097/MD.0000000000025859

**Published:** 2021-06-11

**Authors:** Seon Woo Yoo, Seon Ju Baek, Dong-Chan Kim, A Ram Doo

**Affiliations:** a Department of Anesthesiology and Pain Medicine, Jeonbuk National University Medical School; b Research Institute of Clinical Medicine of Jeonbuk National University-Biomedical Research Institute of Jeonbuk National University Hospital, Jeonju, South Korea.

**Keywords:** malignant hyperthermia, mutation, myotonic dystrophy

## Abstract

**Rationale:**

Several hereditary myopathies that can predispose to malignant hyperthermia (MH) are reported. However, the risk of MH in myotonic dystrophy type I (DM1) has been suggested equal to general population, although the evidence is limited to only a few case reports.

**Patient concerns:**

We encountered a rare case of MH during anesthesia induction with sevoflurane in a male adolescent with previously undiagnosed DM1.

**Diagnoses:**

After the event, genetic testing revealed the presence of a previously unknown heterozygous missense mutation in ryanodine receptor 1 (*RYR1*) associated with MH (c.6898T > C; p.ser2300Pro). Concomitantly, the patient was diagnosed with DM1 with abnormal cytosine-thymine-guanine triplet expansion in the *DMPK* gene.

**Interventions:**

Dantrolene was administered to treat the hypermetabolic manifestations in 20 minutes after the identification of MH.

**Outcomes:**

The patient was successfully treated and discharged without any complications. Laboratory abnormalities were recovered to baseline at postoperative 4 days.

**Lessons:**

The authors suggest that possible MH susceptibility in DM1 patients may be refocused. Genetic testing can be a screening tool for MH susceptibility in these population, prior to receiving general anesthesia.

## 1 Introduction

Malignant hyperthermia (MH) is a fatal pharmacogenetic disorder of skeletal muscle resulting from general anesthesia upon exposure to potent halogenated volatile anesthetics or succinylcholine. It manifests various clinical signs of hypermetabolism including hypercapnia, tachycardia, and generalized muscle rigidity, followed by hyperthermia, metabolic and respiratory acidosis, hyperkalemia, and resultant cardiac arrhythmia or arrest. Dantrolene, the only available therapeutic option for MH, has markedly reduced morbidity and mortality,^[1,2]^ however, it is still a great challenge for physicians to manage MH crisis.

Several neuromuscular diseases strongly associated with MH susceptibility include central core disease, multiminicore myopathy, congenital myopathy with cores and rods, and centronuclear myopathy, all of which are linked to mutations in ryanodine receptor 1 (*RYR1*).^[2–5]^ Myotonic dystrophy type I (dystrophia myotonia type 1, DM1; Steinert disease) is a slowly progressive hereditary muscular disorder characterized by weakness and wasting of involved muscles, usually of the cranial musculature including facial, sternocleidomastoid (SCM), and distal limb muscles. Several studies suggest that the risk of MH is not high in DM1 patients, although the evidence is limited to only a few case reports.^[6–8]^ However, the relationship between several underlying myopathies including DM1 and MH susceptibility is still controversial.^[3,6–9]^ In this case report, we report a MH crisis that developed during inhalation anesthesia with sevoflurane for torticollis surgery in a male adolescent patient with previously undiagnosed DM1.

## 2 Case presentation

**Figure 1:**
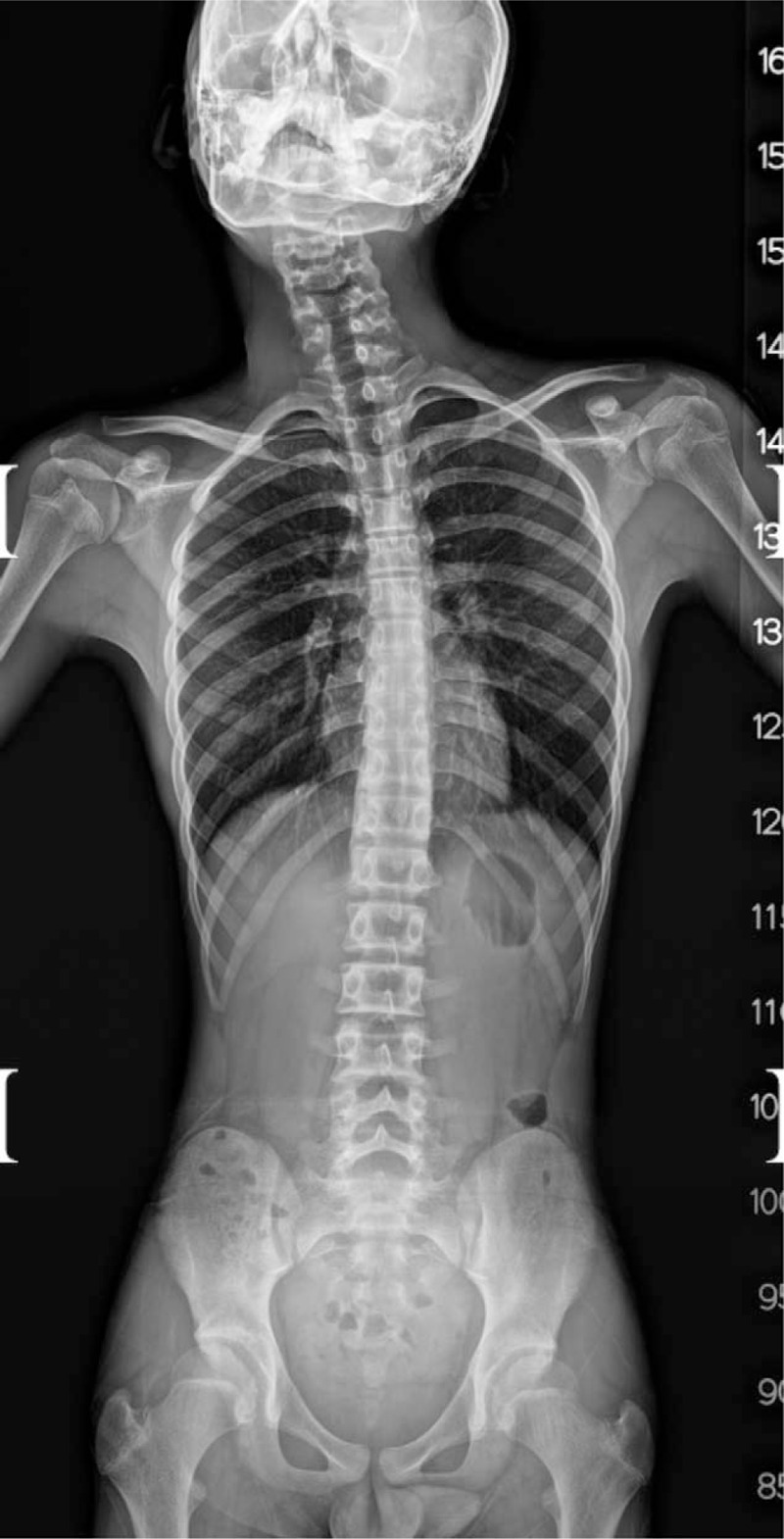
Sternocleidomastoid muscle asymmetry and cervico-thoracic scoliosis.

Written informed consent for publication of this case was obtained from the patient's parents. A 14-year-old male patient was scheduled to undergo SCM muscle release for right-sided muscular torticollis, which was noticed by his parents 2 years earlier. At the preanesthesia evaluation, the patient was previously healthy except for a history of allergic rhinitis, and had no previous history of any anesthetic exposure. He was 157.1 cm in height and 39 kg in weight, and routine laboratory findings including complete blood count, electrolytes, creatinine, chemistry, and coagulation profile were within normal range. Radiologic evaluation of the whole spine revealed SCM asymmetry, in which the volume of the right side was thicker than the left side, and scoliosis in the cervico-thoracic spine (Fig. 1). Psycho-motor development was normal for his age, although he was lying between the 5th and 10th percentiles of the World Health Organization's growth reference.

Upon arriving at the operating room, the baseline blood pressure and heart rate were 113/69 mmHg and 83 beats per minutes (bpm), respectively. Anesthesia was induced with 300 mg of thiopental sodium and maintained with sevoflurane with a mixture of 50% nitrous oxide and oxygen. Rocuronium 30 mg was administered to facilitate endotracheal intubation, and a 6.5-mm cuffed endotracheal tube was placed 20 cm from the central incisors. In 3 minutes, the end-tidal CO_2_ (ETCO_2_) abruptly increased from 36 to over 70 mm Hg. The heart rate increased from 85 to 135 bpm. At the same time, abnormal rigidity in both arms was observed. The nasopharygeal temperature was 36.6 °C and did not increase excessively at this hyperacute stage.

We suspected MH, thus the administration of sevoflurane and nitrous oxide was stopped and the patient was ventilated with 100% oxygen. A new anesthetic circuit and anesthetic machine was introduced to prevent further exposure to the volatile anesthetics. Propofol infusion was started sedate the patient. After removing the patient's clothes, ice packs were applied to the chest and axilla, and cold saline was administered to prevent hyperthermia. For repeated blood gas analysis, an arterial cannula was applied in the radial artery of the patient. A urinary catheter was inserted to monitor diuresis and urine color. Over the course of 15 minutes, increased ETCO_2_ (70–75 mmHg) was sustained despite an increased minute ventilation to 7.7 L/min. The heart rate increased up to 160 bpm. The first arterial gas analysis just before dantrolene administration was as follows: pH = 7.16, PaCO_2_ = 73 mmHg, PaO_2_ = 254 mmHg, lactate = 4.1 mmol/L, and base excess = –4.3 mmol/L. Dantrolene (40 mg, 1 mg/kg; Procter & Gamble Pharmaceuticals, OH) was administered 20 minutes after the identification of MH, and an additional dose of 20 mg was administered subsequently. Sodium bicarbonate (80 mEq) was administered to manage metabolic acidosis.

**Figure 2:**
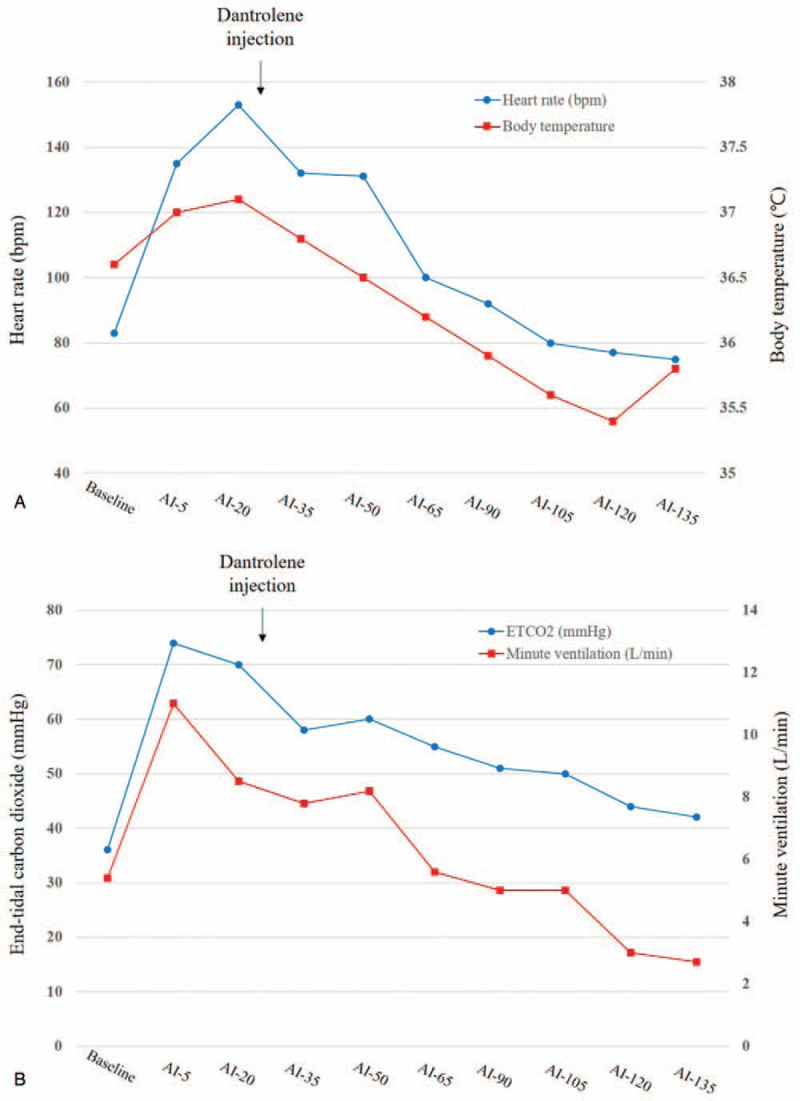
The changes of (A) heart rates and nasopharyngeal temperature. (B) End-tidal carbon dioxide and minute ventilation. AI = anesthesia induction, AI-105 = 105 minutes after AI, AI-120 = 120 minutes after AI, AI-135 = 135 minutes after AI, AI-20 = 20 minutes after AI, AI-35 = 35 minutes after AI, AI-5 = 5 minutes after AI, AI-50 = 50 minutes after AI, AI-65 = 65 minutes after AI, AI-90 = 90 minutes after AI.

Approximately 30 minutes after administration of dantrolene, ETCO_2_ and heart rate decreased to 50 to 55 mm Hg and 80 to 95 bpm, respectively. Although the body temperature increased to 37.1 °C just before administration of dantrolene, it recovered to the preanesthesia level 20 minutes after dantrolene administration. The changes of heart rates, body temperature, ETCO_2_, and minute ventilation supplied during the process are described in Fig. 2A and B. Arterial gas analysis revealed a pH = 7.34, PaCO_2_ = 51 mmHg, PaO_2_ = 327 mmHg, lactate = 2.0 mmol/L and, base excess = 0.9 mmol/L. Plasma myoglobin and creatinine kinase-MB (CK-MB) were excessively increased to 1793 and 11.53 ng/mL, but urine was clear with adequate urine output. Neuromuscular reversal was decided, and neostigmine 1 mg and glycopyrrolate 0.2 mg were administered for residual neuromuscular blockade. The patient was discharged to the intensive care unit (ICU) for close monitoring.

During the 24-hour ICU stay, vital signs including blood pressure, heart rate, oxygen saturation, and ETCO_2_ were maintained within normal range. Tympanic temperature gradually increased again as high as 37.5 °C at 10 hours after MH onset, but it was normalized by passive cooling using ice packs without additional doses of dantrolene. Plasma myoglobin increased to 3000 ng/mL at 2 hours after MH onset and diminished slowly to upper normal range over 4 days. CK-MB increased to 21.91 ng/mL at 10 hours after MH onset and also normalized slowly over 4 days. Plasma creatinine level and estimated glomerular filtration rate were maintained within normal range during the perioperative period. The patient was discharged from the hospital without any complications on the 10th postoperative day.

**Figure 3:**
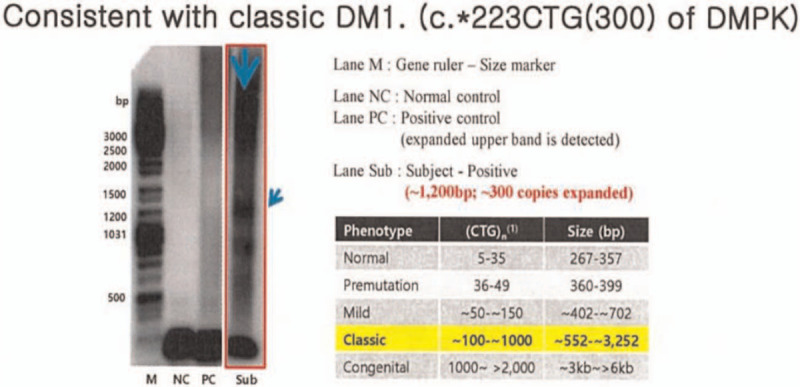
PCR-Southern analysis of *DMPK* on 19q 13.32. The DNA abnormally amplified to 1200 bps, and it means CTG triplet expansion >300 repeats. *DMPK* = dystrophia myotonica-protein kinase. PCR = polymerase chain reaction.

Further evaluations, including genetic analysis and electromyography (EMG), were planned. Genetic testing for the diagnosis of hereditary myopathy as well as MH susceptibility was performed, because a myopathy pattern was shown in EMG. The genetic analysis associated with MH susceptibility revealed a heterozygous missense variant of uncertain significance at *RYR*1 (NM_000540.2 [RYR1]; c.6898T > C, p.ser2300Pro). The *DMPK* (dystrophia myotonica-protein kinase) gene showed at least 300 cytosine-thymine-guanine (CTG) triplet repeats [c.∗223CTG(300)] (Fig. 3). The results are consistent with those of classic DM1. His family members also agreed to perform genetic counseling. They didn’t present any MH susceptibility-associated mutations in the analysis.

## 3 Discussion

MH is an autosomal dominant pharmacogenetic disorder of skeletal muscle. Identifying MH susceptibility is very important for both MH probands and their relatives in preparing for future medical care. In particular, several hereditary myopathies that can predispose to MH susceptibility include central core disease,^[4]^ multiminicore disease,^[5]^ and King–Denborough syndrome.^[10]^ Other myopathies associated with MH-like hypermetabolic reaction (anesthesia-induced rhabdomyolysis; AIR), which is not genuine MH, include DM1 and Duchenne and Becker muscular dystrophy.^[3,7]^ It has been suggested that these patients have an equal risk for MH compared with the general population.^[6–8]^

The differentiation between MH and AIR can be challenging. AIR is characterized by abrupt rhabdomyolysis, hyperkalemia, and resultant cardiac arrest to the exposure to succinylcholine and halogenated anesthetics.^[3]^ The early manifestations for AIR are hyperkalemia-related symptoms, including electrocardiogram changes, cardiac arrhythmias, and arrest. It also presents increased CK level and acidosis in laboratory results, but may rarely induce increased ETCO_2_ and/or temperature increase.^[11]^ However, our patient presented increased ETCO_2_, tachycardia, and muscular rigidity as early manifestations, and serum electrolyte levels were within normal range during the event. According to clinical grading scale by Larach et al,^[12]^ our patient had a score of 58, and the MH rank is 6, which the likelihood of MH is almost certain. Furthermore, hypermetabolic symptoms dramatically diminished following dantrolene administration in our patient, although AIR does not respond to dantrolene.

Molecular genetic testing also support the possibility of MH crisis in DM1 patient in this case. Molecular genetic testing is a recently acceptable method for determining MH susceptibility instead of caffeine-halothane contracture test, a gold standard.^[13]^ The genetic mutations associated with MH susceptibility including *RYR1*,^[14,15]^*CACNA1S*,^[16]^ and *STAC3*^[17]^ had been reported. Above all, the missense mutation of *RYR1*, in which localized on chromosome 19 q13.1, is most frequently detected in MH probands and susceptibility individuals.^[2]^ The identified pathogenic variants of *RYR1* associated with MH susceptibility are expanding the numbers for decades, however, it is not yet fully characterized. Furthermore, advances in molecular and genetic technology of MH concerning the correlation between genotype and phenotype are ongoing.^[18]^ In this case, we report a new heterozygous missense mutation of *RYR1* (c.6898T > C, p.ser2300Pro), which are considered to be associated with MH susceptibility of the patient. Concomitantly, the genetic testing showed 300 repetition or more of Cytosine-Thymine-Guanine (CTG) triplets [c.∗223CTG(300)] in *DMPK* gene, which seems reasonable to assume the MH susceptibility in DM1 patient in this case.

DM1 is a slowly progressive muscular disorder characterized by weakness and wasting of involved muscles, usually of the cranial musculature including facial, SCM, and distal limb muscles. It is an autosomal dominant inherited neuromuscular disease having abnormally expanded CTG repeats in *DMPK* on chromosome 19q 13.3. Because DM1 could involve multisystem throughout the whole body, the preoperative evaluation should be focused on multiple manifestations of the disease. Even though pulmonary complication including acute respiratory failure is most common perioperative complications, gastrointenstinal or cardiac involvement may be problematic perioperatively.^[19–21]^ Moreover, DM1 patients has known to be associated with considerable anesthetic challenge because of the increased sensitivity to hypnotics or analgesics, myotonic reaction to neuromuscular drugs, and inadequate neuromuscular reversal to cholinesterase inhibitors.^[22–25]^ This case report suggests that a patient with DM1 may predispose MH susceptibility, therefore, careful counseling for a personal and family history of MH-like event and genetic evaluation associated to MH susceptibility may be required for anesthetic planning.

The hyper-acute onset of MH is very rare, even though sevoflurane rather than isoflurane, and younger subjects <20 years may manifest faster onset of MH.^[26]^ MH associated with less potent volatile anesthetics such as sevoflurane or desflurane is known to present relatively gradual onset of symptoms.^[27,28]^ Several studies reported that the median MH occurrence time following exposure to sevoflurane without succinylcholine ranged from 45 to 72.5 minutes.^[26,29]^ Our patient presented abrupt manifestation of hypercarbia, tachycardia, and muscular rigidity of both hand as early signs of MH in approximately 5 minutes following beginning of sevoflurane administration. Although temperature increase is an important confirmatory sign for MH, it may develop at the late stage of the MH progress. In our case, the core temperature of the patient maintained within normal range and the patient had only mild temperature rise <37.5 °C until discharge from the ICU. The authors think that the early diagnosis of MH and prompt dantrolene therapy would contribute to the absence of hyperthermia in this patient.

## 4 Conclusion

In conclusion, MH susceptibility in dystrophic myopathies is still inconclusive. We may refocus on the possibility of MH susceptibility in DM1 patients. Genetic testing can be a screening tool for MH susceptibility in these population, prior to receiving general anesthesia.

## Author contributions

**Conceptualization:** Dong-Chan Kim, A Ram Doo.

**Data curation:** Seon Ju Baek.

**Supervision:** Dong-Chan Kim, A Ram Doo.

**Writing – original draft:** Seon Woo Yoo.

**Writing – review & editing:** A Ram Doo.
